# The Trend of C-Reactive Protein After Corticosteroid Therapy in COVID-19 Patients Admitted to IGIMS, Patna

**DOI:** 10.7759/cureus.51499

**Published:** 2024-01-02

**Authors:** Neelima Singh, Randhir Kumar, Shailesh Kumar, Nidhi Prasad, Sweta Muni, Namrata Kumari

**Affiliations:** 1 Infectious Disease, Indira Gandhi Institute of Medical Sciences, Patna, IND

**Keywords:** inflammatory biomarker, cytokine storm syndrome, corticosteroids in covid-19, corona virus disease 2019, crp (c reactive protein)

## Abstract

Background: C-reactive protein (CRP) is a routine inflammation biomarker. Increased CRP levels are correlated with COVID-19. We found a marked reduction in CRP concentration on corticosteroid therapy, which in turn led to reduced mortality and duration of hospital stay.

Methods: In this retrospective cohort study, CRP levels were measured on admission and at 72 hours and compared between two groups of patients, with and without corticosteroid therapy. The study sample consisted of 105 RT-PCR-confirmed patients admitted to the ICU of the COVID ward. Out of the total patients, 57 received one or more doses of dexamethasone in addition to usual treatment, and 48 were given only usual care.

Result: CRP at the time of admission was comparable for both groups. Also, a significant decrease in the CRP was noted in both groups 72 hours post-admission. Moreover, the decline was more marked in the steroid-administered group (CRP-baseline: 34.3mg/dL (+/-8.44), CRP at 72 hours 18.5mg/dL(+/-7.95) (p <0.00) compared to non-steroid group (CRP_baseline: 34.04mg/dL (+/-10.06), CRP at 72. Those with comorbidities were administered steroids (n=38, 66.7%) compared to those who were not (n=08, 16.7%). The average duration of hospital stay was less (5 to 7 days) in the corticosteroid-administered group compared to the other group (7 to 10 days).

Conclusion: Routine CRP tests can predict the outcome and treatment of severe coronavirus disease. Corticosteroid treatment in COVID-19 patients is associated with reduced CRP levels within 72 hours after therapy.

## Introduction

In the latter part of 2019, a zoonotic origin in China first documented the occurrence of COVID-19, initiated by the severe acute respiratory syndrome coronavirus 2 (SARS-CoV-2) [1]. The world, especially India, experienced a significant impact from the second wave of COVID-19 in 2021. India reported more than 300,000 deaths, with 270 million confirmed cases during this wave [2].

The pathophysiology of severe COVID-19 is characterized by an acute pneumonic process, evident through extensive radiologic opacity. Autopsy findings disclose diffuse alveolar damage, inflammatory infiltrates, and microvascular thrombosis [3]. The most severe clinical manifestation encompasses acute respiratory distress syndrome and a robust systemic inflammatory response. A subgroup of individuals exhibits markedly elevated levels of inflammatory markers, including C-reactive protein (CRP), ferritin, interleukin-1, and interleukin-6, contributing to inflammatory organ injury in severe cases [4,5].

Corticosteroids have been proven to significantly and effectively mitigate cytokine storms in some individuals. As per the RECOVERY trial, dexamethasone substantially reduces mortality risk, approximately 33%, particularly in individuals requiring mechanical ventilation [6].

Cytokine storm or cytokine release syndrome is a crucial clinical trait of patients with severe COVID-19. It is distinguished by the release of proinflammatory cytokines and chemokines, which are crucial in the critical and poor prognosis of multiple organ damage and functional failure [7,8]. Through a particular CRP receptor, CRP can increase phagocytosis and help phagocytes eliminate various harmful pathogens [9]. A cytokine response storm that can be set off during COVID-19 pneumonia is linked to a significant fatality rate. A proinflammatory cytokine storm, which occurs in some individuals with severe COVID-19, can exacerbate lung damage and, in certain circumstances, lead to vasodilatory shock and multiorgan failure [10].

The severity and prognosis of exacerbated inflammatory responses in conditions like cardiovascular disease (CVD), type 2 diabetes mellitus (T2DM), hemorrhagic stroke, and sepsis within the context of COVID-19 pneumonia have been notably associated with CRP. CRP, a nonspecific marker of inflammation, plays a dual role as an excellent biomarker for inflammation and a crucial element in disease progression [11].

The key signal for synthesizing CRP is the cytokine IL-6(Interleukin-6), and corticosteroids have been demonstrated to inhibit the production of IL-6 and other cytokines by airway macrophages in vitro and in vivo [12]. It has been demonstrated that CRP levels are correlated with COVID-19 and bacterial pneumonia outcomes [13,14].

C-Reactive Protein level is the direct measure of the acute-phase response that can be used to assess a patient's general level of inflammation. Its concentration changes rapidly, and daily measurements can be useful in the appropriate context [15]. We speculated that prompt corticosteroid administration to COVID-19 patients could mitigate vigorous host respiratory and systemic inflammatory responses, as evidenced by reduced CRP levels.

Aims and objectives

To examine the trend of CRP levels in COVID-19 patients who receive corticosteroid therapy. To compare CRP levels in patients who receive corticosteroids and those who do not.

## Materials and methods

A retrospective cohort study was done in the Virology/Immunology section of the Department of Microbiology at Indira Gandhi Institute of Medical Sciences (IGIMS), Patna. This study was approved by the IGIMS, Patna, Institute Ethics Committee (164/IEC/IGIMS, 2021). The duration of the study was from May 2021 to April 2022. The study encompassed 105 Reverse Transcription-Polymerase Chain reactions (RT-PCR) confirmed COVID-19 patients admitted to COVID-19-designated ICU wards in the hospital. As it is a retrospective study, consent was not obtained from the patient.

Exclusion criteria involved patients who passed away within the initial 48 hours of admission to ensure adequate time for the effects of corticosteroid treatment. Additionally, individuals receiving treatment-dose corticosteroids after 5 days in the hospital course and those on steroid therapy for reasons unrelated to COVID were excluded. The subjects were interviewed for associated comorbidities like diabetes or relevant medical history.

CRP (C-Reactive Protein) levels were measured in the Virology/Immunology section of the Microbiology laboratory of IGIMS. For measurement, 2 ml of blood samples were collected from admitted patients in plain tubes following proper aseptic precautions. The tubes with blood samples were kept at room temperature and then centrifuged at 3000g for 3 to 5 minutes to obtain clear serum above the clotted blood. The readings were acquired through the photometer 5010 by Dyasis Diagnostic System and consumables from Euro Diagnostic Systems Pvt. Ltd.

During sample collection, transportation, and processing, strict COVID-19 protocols were followed to break the chain of transmission. Samples were processed in Biosafety Level 2A cabinets with required safety measures. The study explored trends in CRP levels for patients undergoing corticosteroid treatment compared to those who did not receive such treatment. Examining initial C-reactive protein (CRP) levels over time involved a comprehensive analysis of all patients, comparing those who received corticosteroid treatment with those who did not. Among the corticosteroid-treated group, patients were additionally stratified based on alterations in CRP levels following the initiation of treatment. The baseline for CRP was established as the initial level obtained within the first 48 hours of admission, and for each patient, the last CRP level within 72 hours after commencing treatment was employed to compute the change in CRP level from admission. The change in the quantitative values of C-reactive protein was noted down.

Data analysis

Templates were created in Microsoft Excel sheet (Microsoft, Washington, USA), and statistical analysis was done using SPSS (Statistical Package for Social Sciences) version 27 (IBM, Corporation. Released in 2020, IBM SPSS statistics for Windows version 27.0. Comparison between steroid-administered and non-steroid-administered groups was made based on age, gender, and associated comorbidities using the Mann-Whitney U test (paired t-test). The association between the relevance of steroid therapy and the recovery outcome was done by Fisher's exact test.

## Results

The intervention study was conducted on COVID-19 patients admitted to the ICU ward of a tertiary care hospital. Out of 105 patients included in the study, 57 received at least one dose of dexamethasone in addition to usual care, and 48 were given only usual care. The steroid-administered group had no known contraindication to dexamethasone. All the patients were on some kind of respiratory support, like oxygenation, BiPAP, or mechanical ventilation. The average duration of treatment was 7 to 10 days. The patients were randomly assigned to two groups; one administered steroids, and one did not. The group that was administered steroids belonged to the age group of 41-60 years (n=27, 47.4%), followed by 61-80 years (n=20, 35.1%), while half of them, who were not administered steroids were aged 21-40 years (n=25, 52.1%) followed by two-fifth in the age group 41-60 years (n=20, 41.7%). The two groups were comparable based on gender (p 0.484). Furthermore, those with comorbidities were administered steroids (n=38, 66.7%) compared to those who were not (n=08, 16.7%) (Table 1).

**Table 1:** Comparison between steroid-administered and non-administered groups based on age, gender, and co-morbidities

Features	Steroid-administered (n=57) n,%	Non-steroid administered (n=48) n,%	p-value
Age categories			
21-40	10 (17.5%)	25 (52.1%)	<0.00
41-60	27 (47.4%)	20 (41.7%)	
61-80	20 (35.1%)	03 (6.2%)	
Gender			
Male	33 (57.9%)	31 (64.6%)	0.484
Female	24 (42.1%)	17 (35.4%)	
Co-morbidities			
Present	38 (66.7%)	08 (16.7%)	<0.00
Absent	19 (33.3%)	40 (83.3%)	

**Figure 1:**
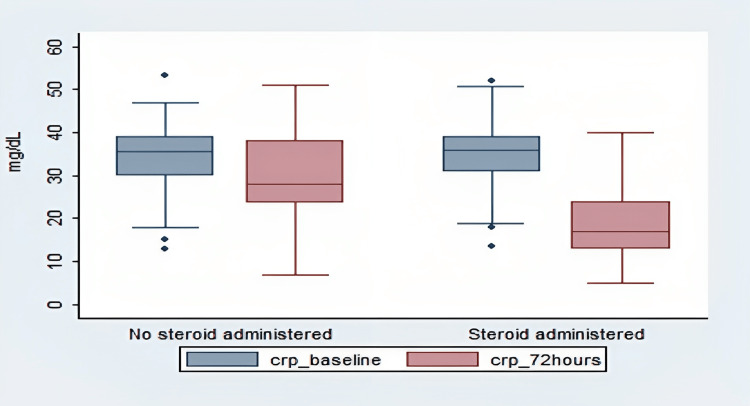
Comparison of difference of CRP in steroid-administered and non-steroid-administered COVID-19 patients CRP: C-reactive protein; crp_b: CRP at admission; crp_72: CRP at 72 hours after admission; 0: Non-steroid-administered; 1: Steroid-administered

It can be deduced from Fig 1 that the CRP at the time of admission was comparable for both groups. Also, a significant decrease in the CRP was noted in both groups 72 hours post-admission. Moreover, the decline was more marked in the steroid- administered group (CRP_baseline: 34.3mg/dL (+/-8.44), CRP at 72 hours 18.5mg/dL(+/-7.95) (p <0.00) compared to non-steroid group (CRP_baseline: 34.04mg/dL (+/-10.06), CRP at 72 hours 18.5mg/dL (+/-7.95) (p 0.0007). Patients with higher CRP levels needed mechanical ventilation.

## Discussion

In this study, it was found that there was a marked reduction in CRP levels in COVID-19 patients following corticosteroid therapy. The group of patients admitted to the ICU who received corticosteroids had better clinical recovery when compared to patients who did not. The factors of age and gender were not found to have any association with CRP levels. Also, those with comorbidities like Diabetes, Cardiovascular disease, Hypertension, etc, were administered steroids (n=38, 66.7%) compared to those who were not (n=08, 16.7%).

CRP, identified as an acute-phase reactant, is manufactured by the liver and can undergo heightened levels in various circumstances like inflammation, cardiovascular disease, and infection [16]. Additional biomarkers associated with unfavorable outcomes, including mortality, severe COVID-19, acute respiratory distress syndrome (ARDS), and the need for intensive care unit (ICU) intervention in COVID-19 patients, comprise Serum Procalcitonin, D-dimer, and Serum Ferritin. CRP binds to phosphocholine found in pathogens and host cell membranes, acting as an opsonin to enhance phagocytosis and contribute to clearance. When CRP is bound to ligands, it also activates the classical pathway of the complement system, a vital component of innate host defense [17]. Elevated CRP concentrations have been observed in bacterial infections, severe viral infections (such as H1N1 influenza pneumonia), and, currently, in SARS-CoV-2 infection [18-21].

The manifestations of COVID-19 encompass a spectrum, spanning from a mild viral infection to a severe acute respiratory illness distinguished by pulmonary inflammation and a subsequent hyper-inflammation phase [4,22]. During the pulmonary phase, there is a progression of dyspnea accompanied by radiographic indications of pneumonia [22]. The early administration of corticosteroids, specifically methylprednisolone, upon the onset of dyspnea may potentially alleviate the advancement to the hyper-inflammation phase.

Our study highlights that in both groups of patients, there is a reduction in CRP levels on serial measurement on admission and at 72 hours, but this decrease in CRP values is markedly significant in the steroid-administered group. Also, the duration of hospital admission days was comparatively less in the administered group. The results of this study are consistent with other research, suggesting that corticosteroids may be an effective treatment to mitigate adverse events in COVID-19 patients who exhibit heightened levels of inflammation, as indicated by CRP levels [6,23,24,25]. The Cochrane Database of Systematic Reviews concludes that systemic corticosteroids likely lead to a slight reduction in all-cause mortality among individuals hospitalized due to symptomatic COVID-19 [26]. However, it is crucial to acknowledge that corticosteroid therapy may be associated with adverse effects in specific patients [23], underscoring the importance of considering alternative treatments. A particular study highlights that patients receiving IL-1 inhibitor therapy showed improved mortality and a significant decrease in CRP concentration compared to a historical group [27]. Additionally, a retrospective study underscores the potential harm associated with corticosteroid therapy in patients with low levels of CRP [6].

Several limitations were present in this study. Being a retrospective study, it has selection bias, and unmeasured confounders may exist. Also, the sample size is smaller, and the study was conducted at a single institution. In an emergency, the second wave of COVID-19 was at its peak, and health-care facilities were constrained.

## Conclusions

With the help of this study, we concluded that prompt corticosteroid treatment in COVID-19 patients is linked to a significant drop in CRP levels within 72 hours after therapy. C-reactive protein (CRP) is a routinely measured biomarker, and reduction in its levels after initiation of therapy may predict inpatient mortality.
